# Supplementary material for: Molecular Characterization of a New Ecotype of Holoparasitic Plant Orobanche L. on Host Weed Xanthium spinosum L
Source: Plants (Basel). 2022 May 25;11(11):1406. doi: 10.3390/plants11111406 (PMC9182829; doi:10.3390/plants11111406)
Supplement: Supplementary file 1 [file plants-11-01406-s001.zip › plants-1695582-supplementary/S2.pdf]

Table S2. Maximum likelihood fits of 24 different models of nucleotide substitution.

Model	BIC	AICc	lnL	Invariant	Gamma	R
K2+G	5929.17	5453.95	-2665.76	n/a	0.84	1.89
K2+I	5931.11	5455.89	-2666.73	0.4	n/a	1.87
K2+G+I	5938.64	5455.64	-2665.6	0.24	2	1.88
T92+G	5941.67	5458.66	-2667.11	n/a	0.83	1.91
T92+I	5942.4	5459.4	-2667.48	0.41	n/a	1.89
T92+G+I	5950.71	5459.92	-2666.74	0.3	2.93	1.9
TN93+I	5951.86	5445.51	-2657.52	0.4	n/a	1.94
HKY+I	5954.17	5455.6	-2663.57	0.41	n/a	1.9
GTR+I	5962.66	5432.96	-2648.22	0.41	n/a	1.85
GTR+G	5969.92	5440.22	-2651.85	n/a	0.86	1.64
K2	5972.2	5504.76	-2692.18	n/a	n/a	1.77
TN93+G	5977.27	5470.91	-2670.22	n/a	0.84	1.95
GTR+G+I	5977.91	5440.42	-2650.94	0.34	5.33	1.62
HKY+G	5978.88	5480.31	-2675.92	n/a	0.83	1.91
T92	5985.46	5510.24	-2693.91	n/a	n/a	1.77
TN93+G+I	5986.26	5472.12	-2669.81	0.3	3.16	1.94
HKY+G+I	5987.98	5481.63	-2675.57	0.31	3.19	1.91
TN93	5994.6	5496.03	-2683.78	n/a	n/a	1.8
HKY	5997.34	5506.56	-2690.05	n/a	n/a	1.79
GTR	6009.47	5487.55	-2676.52	n/a	n/a	1.77
JC+G	6057.91	5590.47	-2735.03	n/a	0.91	0.5
JC+I	6059.71	5592.27	-2735.93	0.39	n/a	0.5
JC+G+I	6067.37	5592.15	-2734.86	0.23	2.12	0.5
JC	6096.12	5636.47	-2759.04	n/a	n/a	0.5

GTR: General Time Reversible; HKY: Hasegawa-Kishino-Yano; TN93: Tamura-Nei; T92: Tamura 3-parameter; K2: Kimura 2-parameter; JC: Jukes-Cantor; R: estimated transition/transversion bias.
