# Supplementary material for: Molecular Characterization of a New Ecotype of Holoparasitic Plant Orobanche L. on Host Weed Xanthium spinosum L
Source: Plants (Basel). 2022 May 25;11(11):1406. doi: 10.3390/plants11111406 (PMC9182829; doi:10.3390/plants11111406)
Supplement: Supplementary file 1 [file plants-11-01406-s001.zip › plants-1695582-supplementary/S3.pdf]

Table S3. Test of the homogeneity of substitution patterns between sequences. Numbers are the same as in Table 1.

	1	2	3	4	5	6	7	8	9	10	11	12	13	14	15	16	17	18	19	20	21	22	23	24	25	26	27	28	29	30
2	0.000																													
3	0.000	1.000																												
4	0.000	1.000	1.000																											
5	0.000	1.000	1.000	1.000																										
6	0.000	1.000	1.000	1.000	1.000																									
7	0.000	0.072	0.072	1.000	0.264	1.000																								
8	0.000	1.000	1.000	1.000	1.000	1.000	1.000																							
9	0.000	1.000	1.000	1.000	1.000	1.000	1.000	1.000																						
10	0.000	1.000	1.000	1.000	1.000	1.000	1.000	1.000	1.000																					
11	0.000	1.000	1.000	1.000	0.092	1.000	0.292	0.284	1.000	1.000																				
12	0.000	1.000	1.000	1.000	0.112	1.000	0.244	0.224	1.000	1.000	1.000																			
13	0.000	1.000	1.000	0.432	0.438	0.434	0.100	0.244	0.312	1.000	1.000	1.000																		
14	0.000	1.000	1.000	1.000	1.000	1.000	0.238	1.000	1.000	1.000	0.090	0.094	0.418																	
15	0.000	1.000	1.000	1.000	0.080	1.000	0.236	0.224	1.000	1.000	1.000	1.000	1.000	0.054																
16	0.000	1.000	1.000	1.000	1.000	1.000	0.330	1.000	1.000	1.000	0.416	0.378	1.000	1.000	0.420															
17	0.000	1.000	1.000	0.236	0.268	0.240	0.058	0.112	0.352	1.000	1.000	1.000	1.000	0.288	1.000	1.000														
18	0.000	0.338	0.092	1.000	0.250	1.000	1.000	1.000	0.274	1.000	0.438	0.450	0.250	0.224	0.404	0.146	0.164													
19	0.000	0.102	0.082	0.094	1.000	0.086	0.012	0.282	1.000	0.362	0.108	0.110	0.108	1.000	0.088	1.000	0.206	0.048												
20	0.000	1.000	0.308	1.000	1.000	1.000	0.224	0.312	0.296	1.000	1.000	1.000	1.000	1.000	1.000	0.302	1.000	0.238	0.232											
21	0.000	0.000	0.002	0.002	0.014	0.002	0.000	0.004	0.006	0.008	0.000	0.002	0.010	0.006	0.002	0.012	0.004	0.000	0.014	0.000										
22	0.000	0.078	0.088	0.044	0.078	0.028	0.032	0.048	0.040	0.046	0.070	0.068	0.068	0.054	0.056	0.084	0.114	0.034	0.084	0.120	0.266									
23	0.000	0.198	0.186	0.174	0.208	0.178	0.120	0.148	0.186	0.158	0.176	0.174	0.262	0.196	0.212	0.194	0.232	0.086	0.212	0.266	0.316	0.266								
24	0.004	1.000	1.000	0.344	1.000	0.322	0.260	0.338	0.354	0.404	1.000	1.000	1.000	1.000	1.000	1.000	1.000	0.278	1.000	1.000	0.036	0.368	1.000							
25	0.008	1.000	1.000	1.000	1.000	1.000	0.350	1.000	1.000	1.000	1.000	1.000	1.000	1.000	1.000	1.000	1.000	0.362	1.000	1.000	0.094	0.346	1.000	1.000						
26	0.000	1.000	0.238	1.000	1.000	1.000	1.000	1.000	0.300	1.000	1.000	1.000	1.000	1.000	1.000	0.230	1.000	1.000	0.202	1.000	0.004	0.058	0.176	1.000	1.000					
27	0.000	0.174	0.184	0.140	0.218	0.152	0.098	0.138	0.114	0.144	0.166	0.156	0.220	0.210	0.172	0.222	0.190	0.112	0.212	0.254	0.334	0.102	1.000	1.000	1.000	0.184				
28	0.000	0.176	0.182	0.110	0.190	0.156	0.082	0.152	0.144	0.160	0.174	0.144	0.184	0.154	0.170	0.214	0.230	0.074	0.202	0.262	0.230	0.350	1.000	1.000	1.000	0.210	1.000			
29	0.000	1.000	1.000	1.000	1.000	1.000	1.000	1.000	1.000	1.000	1.000	1.000	1.000	1.000	1.000	1.000	1.000	1.000	1.000	1.000	0.006	0.146	0.318	1.000	1.000	1.000	0.310	0.368		
30	0.000	0.196	0.206	0.168	0.200	0.146	0.088	0.162	0.164	0.168	0.170	0.178	0.212	0.192	0.184	0.256	0.208	0.116	0.252	0.234	1.000	0.136	1.000	0.388	1.000	0.180	1.000	1.000	0.260	
31	0.000	0.110	0.106	0.078	0.094	0.070	0.040	0.080	0.094	0.094	0.112	0.132	0.142	0.096	0.114	0.128	0.148	0.054	0.134	0.172	0.218	1.000	1.000	1.000	1.000	0.108	0.264	1.000	0.274	0.242

The probability of rejecting the null hypothesis that sequences have evolved with the same pattern of substitution, as judged from the extent of differences in base composition biases between sequences (Disparity Index test). A Monte Carlo test (500 replicates) was used to estimate the P-values, which are shown bottom the diagonal. P-values smaller than 0.05 are considered significant (marked with yellow highlights). The estimates of the disparity index per site are shown for each sequence pair below the diagonal. The analysis involved 31 nucleotide sequences.
