# Supplementary material for: Molecular Characterization of a New Ecotype of Holoparasitic Plant Orobanche L. on Host Weed Xanthium spinosum L
Source: Plants (Basel). 2022 May 25;11(11):1406. doi: 10.3390/plants11111406 (PMC9182829; doi:10.3390/plants11111406)
Supplement: Supplementary file 1 [file plants-11-01406-s001.zip › plants-1695582-supplementary/S1.pdf]

Table S1. Estimates of evolutionary divergence between sequences

	1	2	3	4	5	6	7	8	9	10	11	12	13	14	15	16	17	18	19	20	21	22	23	24	25	26	27	28	29	30
2	0.058																													
3	0.058	0.000																												
4	0.060	0.002	0.002																											
5	0.060	0.002	0.002	0.003																										
6	0.056	0.002	0.002	0.003	0.003																									
7	0.062	0.003	0.003	0.005	0.005	0.005																								
8	0.062	0.003	0.003	0.005	0.005	0.005	0.007																							
9	0.062	0.003	0.002	0.005	0.005	0.005	0.007	0.007																						
10	0.058	0.003	0.003	0.005	0.005	0.005	0.007	0.007	0.007																					
11	0.060	0.002	0.002	0.003	0.003	0.003	0.005	0.005	0.005	0.005																				
12	0.060	0.002	0.002	0.003	0.003	0.003	0.005	0.005	0.005	0.005	0.003																			
13	0.060	0.002	0.002	0.003	0.003	0.003	0.005	0.005	0.005	0.005	0.003	0.003																		
14	0.060	0.002	0.002	0.003	0.003	0.003	0.005	0.005	0.005	0.005	0.003	0.003	0.003																	
15	0.060	0.002	0.002	0.003	0.003	0.003	0.005	0.005	0.005	0.005	0.003	0.003	0.003	0.003																
16	0.066	0.009	0.007	0.011	0.011	0.011	0.012	0.012	0.009	0.012	0.011	0.011	0.010	0.011	0.011															
17	0.062	0.003	0.003	0.005	0.005	0.005	0.007	0.007	0.007	0.007	0.005	0.005	0.005	0.005	0.005	0.012														
18	0.068	0.009	0.007	0.011	0.011	0.011	0.012	0.012	0.009	0.012	0.011	0.011	0.010	0.011	0.011	0.014	0.012													
19	0.060	0.003	0.003	0.005	0.005	0.005	0.007	0.007	0.007	0.007	0.005	0.005	0.005	0.005	0.005	0.010	0.007	0.012												
20	0.068	0.010	0.009	0.012	0.012	0.012	0.014	0.014	0.010	0.014	0.012	0.012	0.012	0.012	0.012	0.016	0.014	0.016	0.014											
21	0.364	0.271	0.272	0.275	0.268	0.275	0.278	0.271	0.278	0.278	0.268	0.275	0.274	0.275	0.268	0.274	0.275	0.285	0.274	0.286										
22	0.365	0.288	0.284	0.292	0.284	0.292	0.288	0.296	0.287	0.295	0.292	0.292	0.291	0.292	0.284	0.283	0.292	0.295	0.290	0.296	0.196									
23	0.348	0.262	0.259	0.266	0.258	0.266	0.262	0.269	0.261	0.269	0.266	0.266	0.265	0.266	0.258	0.258	0.266	0.269	0.264	0.269	0.163	0.044								
24	0.329	0.252	0.253	0.256	0.256	0.256	0.252	0.259	0.259	0.259	0.256	0.256	0.255	0.256	0.249	0.255	0.256	0.266	0.254	0.267	0.183	0.088	0.062							
25	0.413	0.320	0.316	0.324	0.315	0.324	0.324	0.328	0.319	0.327	0.324	0.324	0.323	0.324	0.315	0.314	0.324	0.319	0.322	0.328	0.225	0.173	0.172	0.201						
26	0.068	0.009	0.007	0.010	0.010	0.010	0.012	0.012	0.009	0.012	0.010	0.010	0.010	0.010	0.010	0.014	0.012	0.014	0.012	0.012	0.275	0.284	0.266	0.263	0.324					
27	0.338	0.254	0.251	0.258	0.250	0.258	0.254	0.261	0.253	0.260	0.258	0.258	0.257	0.258	0.250	0.250	0.258	0.260	0.256	0.261	0.183	0.046	0.027	0.069	0.172	0.258				
28	0.298	0.226	0.226	0.229	0.229	0.229	0.226	0.233	0.232	0.232	0.229	0.229	0.229	0.229	0.223	0.229	0.229	0.239	0.228	0.239	0.151	0.056	0.042	0.073	0.164	0.236	0.046			
29	0.124	0.058	0.056	0.060	0.060	0.060	0.062	0.062	0.058	0.062	0.060	0.060	0.060	0.060	0.060	0.064	0.060	0.064	0.058	0.062	0.259	0.290	0.259	0.264	0.298	0.058	0.255	0.247		
30	0.355	0.268	0.265	0.271	0.264	0.271	0.268	0.275	0.267	0.274	0.271	0.271	0.271	0.271	0.264	0.263	0.271	0.274	0.270	0.275	0.173	0.040	0.018	0.058	0.161	0.271	0.027	0.042	0.261	
31	0.368	0.291	0.288	0.295	0.287	0.295	0.291	0.299	0.290	0.298	0.295	0.295	0.294	0.295	0.287	0.286	0.295	0.298	0.293	0.299	0.193	0.005	0.042	0.086	0.170	0.287	0.044	0.054	0.285	0.038

The number of base substitutions per site from between sequences are shown. Analyses were conducted using the Kimura 2-parameter model. The rate variation among sites was modeled with a gamma distribution (shape parameter = 0.88). The analysis involved 31 nucleotide sequences. All positions with less than 95% site coverage were eliminated. That is, fewer than 5% alignment gaps, missing data, and ambiguous bases were allowed at any position. There were a total of 581 positions in the final dataset.
